# Evolved α‐factor prepro‐leaders for directed laccase evolution in *Saccharomyces cerevisiae*

**DOI:** 10.1111/1751-7915.12838

**Published:** 2017-08-14

**Authors:** Ivan Mateljak, Thierry Tron, Miguel Alcalde

**Affiliations:** ^1^ Department of Biocatalysis Institute of Catalysis CSIC Cantoblanco 28049 Madrid Spain; ^2^ Aix Marseille Université, Centrale Marseille, CNRS, iSm2 UMR 7313 13397 Marseille France

## Abstract

Although the functional expression of fungal laccases in *Saccharomyces cerevisiae* has proven to be complicated, the replacement of signal peptides appears to be a suitable approach to enhance secretion in directed evolution experiments. In this study, twelve constructs were prepared by fusing native and evolved α‐factor prepro‐leaders from *S. cerevisiae* to four different laccases with low‐, medium‐ and high‐redox potential (PM1L from basidiomycete PM1; PcL from *Pycnoporus cinnabarinus*; TspC30L from *Trametes* sp. strain C30; and MtL from *Myceliophthora thermophila*). Microcultures of the prepro‐leader:laccase fusions were grown in selective expression medium that used galactose as both the sole carbon source and as the inducer of expression so that the secretion and activity were assessed with low‐ and high‐redox potential mediators in a high‐throughput screening context. With total activity improvements as high as sevenfold over those obtained with the native α‐factor prepro‐leader, the evolved prepro‐leader from PcL (α^PcL^) most strongly enhanced secretion of the high‐ and medium‐redox potential laccases PcL, PM1L and TspC30L in the microtiter format with an expression pattern driven by prepro‐leaders in the order α^PcL^ > α^PM^ ^1L^ ~ α^native^. By contrast, the pattern of the low‐redox potential MtL was α^native^ > α^PcL^ > α^PM^ ^1L^. When produced in flask with rich medium, the evolved prepro‐leaders outperformed the α^native^ signal peptide irrespective of the laccase attached, enhancing secretion over 50‐fold. Together, these results highlight the importance of using evolved α‐factor prepro‐leaders for functional expression of fungal laccases in directed evolution campaigns.

## Introduction

Fungal laccases (EC 1.10.3.2, benzenediol:oxygen oxidoreductases) catalyse the oxidation of phenols, aromatic amines and other compounds, with the concomitant reduction of molecular oxygen to water (Solomon *et al*., 1996; Gianfreda *et al*., 1999; Alcalde, 2007). The laccase substrate spectrum can be expanded notably through the laccase mediator system, a system based on diffusible electron carriers that become strong oxidizers upon oxidation by laccase to act then on other substrates – mostly non‐phenolics – that are otherwise little oxidized by the laccase alone (Morozova *et al*., 2007; Cañas and Camarero, 2010). Given this broad substrate range and their minimal requirements, fungal laccases belong to the elite of oxidases that can be employed in very distinct areas of biotechnology, from organic synthesis to novel green processes and beyond (Riva, 2006; Kunamneni *et al*., 2008a,b; Mate and Alcalde, 2017). For decades, these blue multicopper‐containing enzymes have attracted much interest and as such, they have been the focus of many attempts to engineer them through directed evolution with a view to adapt them to harsh industrial conditions, making them resistant to high temperature or extreme pH, or functional in the presence of different types of inhibitors or organic solvents, to name but a few (Rodgers *et al*., 2010; Mate and Alcalde, 2015, 2016). Assisted by a strong portfolio of solutions that combine bio‐ and electro‐catalysis, the application of engineered laccases is no longer a pipedream. However, this new age of directed laccase evolution requires tools and library creation methods that can be readily manipulated to help generate superior biocatalysts.

One of the main hurdles when engineering fungal laccases is their poor functional expression in heterologous hosts and limited secretion. Due to its eukaryotic nature and simple fermentation requirements, *Saccharomyces cerevisiae* is a suitable microorganism to improve recombinant laccases by directed evolution (Gonzalez‐Perez *et al*., 2012). With an efficient DNA recombination apparatus, this yeast allows us to perform a wide array of genetic manipulations, facilitating the generation of molecular diversity. Protein engineering strategies have been used to boost laccase secretion in *S. cerevisiae*, including (i) the replacement of the native signal peptide with different prepro‐leaders, (ii) directed evolution of the mature laccase, (iii) directed evolution of prepro‐leaders, and (iv) a combination of these approaches.

The evolution of α‐factor prepro‐leaders from *S. cerevisiae* is exceptionally relevant, in the hope that they could serve as universal signal peptides in different directed laccase evolution enterprises, an issue that has yet to be addressed. The pioneering work of the Wittrup group indicated that directed evolution of α‐factor prepro‐leaders could enhance the expression of different types of proteins in yeast, from full‐length antibodies to cellulases (Rakestraw *et al*., 2009; Dana *et al*., 2012). However, when we have tested evolved α‐factor prepro‐leaders in different groups of ligninases (e.g. evolved prepro‐leaders from laccases to enhance the secretion of unspecific peroxygenases (Molina‐Espeja *et al*., 2014)), the results were not encouraging, suggesting that evolved prepro‐leaders may only be successfully exchanged between proteins of similar phylogeny. Conversely, it still remains unclear whether an α‐factor prepro‐leader that has been evolved to enhance protein expression can be translated to a different enzyme group to achieve similar benefits or can be even effectively transferred between proteins that belong to the same enzyme group. Particularly, the use of evolved prepro‐leaders for directed laccase evolution experiments could help enhance secretion levels in high‐throughput screening – HTS – format (i.e. cultures in microtiter plates). Should this be the case, the oxidation of high‐redox potential mediators that are barely oxidized by laccase might be readily detected during screening such that their oxidation rates could be improved by iterative rounds of directed evolution.

In this study, we combined different native and evolved prepro‐leaders from previous directed evolution campaigns with four fungal laccases that display low‐, medium‐ and high‐redox potential and a protein sequence identity between 26 and 73%. Twelve α‐factor prepro‐leader:laccase fusions were constructed and their influence on expression and secretion was assessed in HTS format with low‐ and high‐redox potential mediators (2,2’‐azino‐bis(3‐ethylbenzothiazoline‐6‐sulphonic acid (ABTS) and K_4_[Mo(CN)_8_] respectively) so that the restricted growth conditions of a directed evolution round in terms of poor cell growth and enzyme secretion were emulated. A secretion pattern driven by the prepro‐leader attached to the laccase was established and discussed within a mutational context.

## Results and discussion

**Figure 1:**
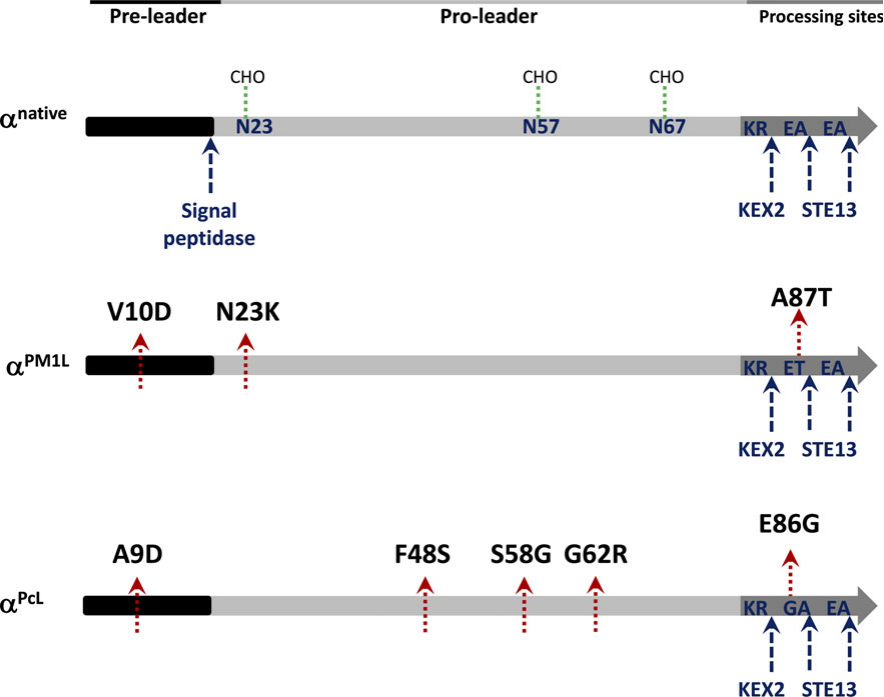
Native and evolved α‐factor prepro‐leaders. Processing sites in the pre‐ and pro‐regions are indicated by the blue arrows, the red arrows highlight mutations and the green dotted lines indicate glycosylation sites. α^native^, native α‐factor prepro‐leader; α^PcL^, evolved α‐factor prepro‐leader from a previous evolution campaign performed on PcL (Camarero *et al*., 2012); α^PM^ ^1L^, evolved α‐factor prepro‐leader from a previous evolution campaign performed on PM1L (Mate *et al*., 2010). The α‐factor prepro‐leader:laccase fusions were constructed by gene assembly through *in vivo* overlap extension (IVOE; Alcalde, 2010). All PCR reactions were cleaned, concentrated, loaded in preparative low melting point agarose gels (0.75% w:v) and purified. The constructs were cloned under the control of the GAL1 promoter of the pJRoC30 expression shuttle vector, which was linearized with BamHI and XhoI, and the linear plasmid was concentrated and purified as above. The reaction mixtures contained DNA template (10 ng μl^−1^), 1 mM dNTPs (0.25 mM each), 3% (v/v) dimethylsulfoxide (DMSO) and 0.05 U/of Pfu Ultra DNA polymerase in a final volume of 50 μl, along with the appropriate primers (0.25 μM). The design of the overlapping 40 bp regions between adjacent fragments allowed the homologous recombination machinery of *S. cerevisiae* to drive the *in vivo* fusion and cloning of the different genetic elements (protease‐deficient *S. cerevisiae* strain BJ5465).

The α‐factor prepro‐leader from *S. cerevisiae* is classically employed to enhance the secretion of foreign proteins by yeast (Shuster, 1991; Romanos *et al*., 1992). This secretory leader contains a pre‐region of 19 amino acids and a pro‐region of 64 amino acids with three N‐linked glycosylation sites, Fig. 1. The canonical pre‐leader is implicated in the translocation of the nascent secretory protein, which is removed from the endoplasmic reticulum (ER) membrane by the action of a signal peptidase between residues 19 and 20. At this point a primary oligosaccharide is added, after which the protein is packed into vesicles for transportation to the Golgi where it is further glycosylated by long outer chains of mannose residues. The α‐factor pro‐leader is thought to display chaperone‐like activity, and it is processed in the Golgi compartment through the action of KEX2, STE13 and KEX1 proteases (the latter of which is unnecessary for the heterologous expression of α‐factor prepro‐leader fusion proteins).

Some years ago, our laboratory achieved the heterologous functional expression in *S. cerevisiae* of two different high‐redox potential laccases from basidiomycete PM1 (PM1L) and *Pycnoporus cinnabarinus* (PcL; Mate *et al*., 2010; Camarero *et al*., 2012). After attaching them to the native α‐factor prepro‐leader, these fusions were subjected to joint rounds of directed evolution to improve secretion. Similarly, we were also involved in the directed evolution of the low‐redox potential laccase from the ascomycete *Myceliophthora thermophila* (MtL). In this case, the laccase, as well as its native prepro‐leader and C‐terminal – which was successfully processed after introducing a KEX2 cleavage site, were evolved together (Bulter *et al*., 2003). In the current work, the native *S. cerevisiae* α‐factor prepro‐leader and the evolved α‐factor prepro‐leaders from PM1L and PcL (α^native^, α^PM1L^ and α^PcL^ respectively) were tested to explore their possible combination with evolved laccase mutants PM1, PcL and MtL, and also with the native laccase isoform LAC3 from the basidiomycete *Trametes* sp. strain C30 (TspC30L), which has proved to be heterologously expressed by yeast (Klonowska *et al*., 2005), Fig. 1. The protein sequence identity between these four laccases ranges from 73% to 26%, where three of the four laccases (TspC30, PM1L and PcL) are medium‐ to high‐redox potential laccases with a sequence identity window of 69–73% at the amino acid level, Table 1. Accordingly, twelve α‐factor prepro‐leader:laccase fusions were generated by gene assembly through IVOE (Alcalde, 2010), and secretion was assessed within a HTS context so that conditions found during a directed evolution experiment were rapidly reproduced.

**Table 1:** Laccase used in the present study

Laccase	E°T1 (mV)	Amino acids	Alignment	Score (%)
aPcL	+790	497	PcL:PM1L	72.98
			PcL:TspC30L	68.81
			PcL:MtL	30.38
bPM1L	+760	496	PM1L:TspC30L	70.16
			PM1L:MtL	28.83
cTspC30L	+680	501	TspC30L:MtL	25.75
dMtL	+475	559		

a PcL: evolved mutant 3PO with the mutations V162A, H208Y, S224G, A239P, D281E, S426N and A461T in the mature protein (Camarero *et al*., 2012).

b PM1L: evolved mutant OB‐1 with the mutations N208S, R280H, N331K, D341N and P394H in the mature protein (Mate *et al*., 2010).

c TspC30L: native laccase isoform LAC3 from Trametes sp. C30.

d MtL: evolved mutant T2 with mutations S3I, E86G, A108V, N303S, F351L, T366M, Y403H, S450P, N454K, L536F, Y552N, H(C2)R (Bulter *et al*., 2003).

**Figure 2:**
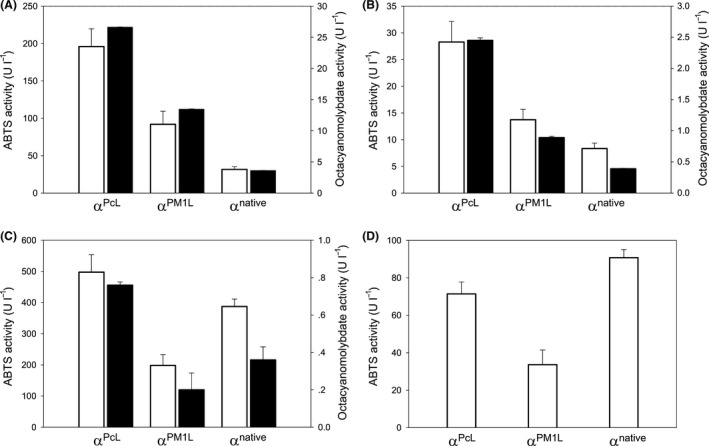
Laccase secretion in SEM under the HTS format. (A) PM1L; (B) PcL; (C) TspC30L; (D) MtL; α^native^, native α‐factor prepro‐leader from *S. cerevisiae*; α^PcL^, evolved α‐factor prepro‐leader from a previous evolution campaign performed on PcL (Camarero *et al*., 2012); α^PM^ ^1L^, evolved α‐factor prepro‐leader from a previous evolution campaign performed on PM1L (Mate *et al*., 2010); white bars, total activity measured with ABTS; black bars, total activity measured with K_4_[Mo(CN)_8_]. Measurements were obtained from eight independent microcultures and expressed as the mean plus standard deviation. Selective expression medium (SEM) contained 100 ml yeast nitrogen base 67 g l^−1^, 100 ml yeast synthetic dropout medium without uracil 19.2 g l^−1^, 100 ml galactose 20%, 67 ml KH_2_ PO_4_ buffer 1 M [pH 6.0], 31.6 ml ethanol 100%, 1 ml CuSO_4_ 1 M, 1 ml chloramphenicol 25 g l^−1^ and sterile double‐distilled H_2_O (*sdd*H_2_O) to 1000 ml. Individual clones of the laccase constructs were picked and cultured in sterile 96‐well plates containing 200 μl of SEM. The plates were sealed to prevent evaporation and incubated for 72 h at 30°C in a humidity shaker at 225 rpm and 80% relative humidity (Minitron‐INFORS; Biogen, Spain). The plates (master plates) were centrifuged for 15 min at 3000 rpm at 4°C (Eppendorf 5810R centrifuge with A‐4‐62 rotor, Germany), and 20 μl of supernatant was transferred (with the help of a robot Liquid Handler EVOFreedom‐100, TECAN, Switzerland) into two replica plates: ABTS activity plate and K_4_[Mo(CN)_8_] activity plate. The corresponding reaction mixture was then added to each plate (180 μl) using a Multidrop robot (Multidrop Combi, Thermo Fischer Scientific, Vantaa, Finland). The reaction mixture for ABTS plates contained 100 mM citrate–phosphate buffer (pH 4.0) and 3 mM ABTS, while that for the K_4_[Mo(CN)_8_] plates contained 100 mM citrate–phosphate buffer (pH 4.0) and 2 mM K_4_[Mo(CN)_8_]. The plates were stirred briefly, and the absorption at 418 nm (ε_ABTS_˙^+^ = 36 000 M^−1^ cm^−1^) was recorded in kinetic mode on a microplate reader (SpectraMax Plus 384, Molecular Devices, Sunnyvale, CA), or at 388 nm (ε_K3Mo(_ _CN__)8_ = 1460 M^−1^ cm^−1^) for K_4_Mo(CN)_8_ oxidation. To rule out false positives, two consecutive re‐screenings were carried out, as reported elsewhere (Mate *et al*., 2010).

It is worth noting that cell growth in HTS microculture format is far from ideal (in terms of oxygen availability and stirring limitation), implying severe constrains during the preculture, growth and production phases. Although the use of a rich non‐selective medium is preferred in the final stages of larger fermentations, it is not always suitable to produce laccase mutant libraries in an HTS format as it may interfere with the screening of different high‐redox potential mediators whose oxidized products could yield responses at the UV/VIS wavelength frontier (unpublished material). Moreover, the secretion of native proteins and ancillary factors by the yeast may also affect the measurements. Therefore, a selective expression medium (SEM) for laccase secretion by *S. cerevisiae* in HTS format was used to overcome these hurdles. This SEM contained a supplement of copper to favour cofactor uptake by laccases and more importantly, galactose (instead of raffinose or glucose) as the only carbon source to trigger laccase expression under the control of the GAL1/10 promoters (see legend for Fig. 2). SEM allowed laccase activity to be measured at both the near UV and visible wavelengths while providing resistance against plasmid degradation given that selection is exerted during all growth stages.

In terms of the screening assays, two different redox mediators were chosen, each with a E° that is pH‐independent: ABTS, E°_ABTS_^•+^ = +690 versus NHE; K_4_[Mo(CN)_8_], E° = +780 mV versus NHE. ABTS is a mediator whose radical cation ABTS^•+^ gives a reliable colorimetric response with a maximum of absorbance ~418 nm. This organic molecule is becoming a common substrate for HTS assays in different evolution campaigns involving laccases, peroxidases and peroxygenases (Alcalde, 2015). By contrast, K_4_[Mo(CN)_8_] is a mediator with a higher redox potential that belongs to the group of transition metal coordination complexes and it can cycle between ‐4/‐3 redox states. As such, K_4_[Mo(CN)_8_] does not yield a radical product upon oxidation by laccase as the electron exchange is focused on the metallic atom of the complex but it does follow an electron transfer route, as ABTS (Rochefort *et al*., 2004). While its reaction product gives reliable response at 388 nm, K_4_[Mo(CN)_8_], like other high‐redox potential mediators, is hardly oxidized by low‐redox potential laccases. Therefore, detection of K_4_[Mo(CN)_8_] oxidation in HTS format is complicated unless large quantities of laccase are secreted into the medium.

Under these premises, the secretion of α‐factor prepro‐leader:laccase fusions grown in SEM/HTS format was evaluated using ABTS and K_4_[Mo(CN)_8_]. Notably, when PM1L was fused to the evolved α‐factor prepro‐leader from PcL (α^PcL^, Fig. 1), secretion augmented ~7‐fold irrespective of the redox mediator tested, Fig. 2A. Similar results were obtained with PcL fusions, although the total activity detected in the microculture broth was less than that of the PM1L fusions due to their weaker expression (2 and 8 mg l^−1^ for PcL and PM1L mutants respectively; Camarero *et al*., 2012; Mate *et al*., 2010), Fig. 2B. Thus, evolved α‐factor prepro‐leaders conferred a similar pattern of secretion to the high‐redox potential laccases PM1L and PcL, in the order α^PcL^ > α^PM1L^ > α^native^. The strongest secretion of the medium‐redox potential TspC30L was also achieved when fused to α^PcL^ (with a production of ~500 ABTS U l^−1^ and a secretion pattern α^PcL^ > α^native^ > α^PM1L^) despite the fact that this prepro‐leader was originally evolved for PcL. By contrast, secretion of the low‐redox potential MtL was similar for both the α^native^ and α^PcL^ constructions, Fig. 2C and D. Thus, the strong correlation between protein sequence identity and secretion driven by the different prepro‐leaders indicates that while the secretion of medium‐ and high‐redox potential laccases (with a sequence identity in the range 69–73%) can be improved by attaching them to α‐factor prepro‐leaders evolved for their functional expression, MtL – which shares 26–30% sequence identity with its laccase counterparts – does not follow the same rules, at least within a HTS format (see below).

The oxidation of K_4_[Mo(CN)_8_] was followed readily in the HTS context for both PM1L and PcL, the latter displaying lower responses due to its more limited secretion. As expected, the medium‐redox potential TspC30L also gave a reliable response with this substrate, albeit to a much lesser extent than its high‐redox potential laccase counterparts, Fig. 2A–C. Finally, no activity was recorded with the low‐redox potential MtL, irrespective of the fusion tested, Fig. 2D. These results highlight the benefits of combining K_4_[Mo(CN)_8_] with SEM for evolving and/or searching high‐redox potential laccases.

**Figure 3:**
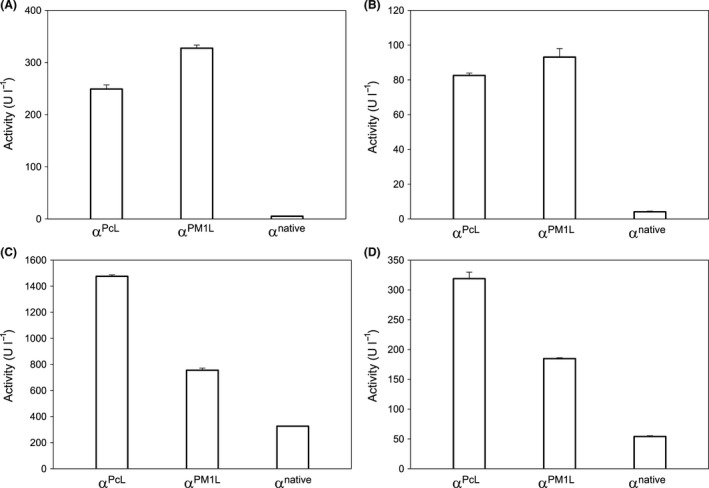
Laccase secretion in rich medium with flask fermentation. (A) PM1L; (B) PcL; (C) TspC30L; (D) MtL; α^native^, native α‐factor prepro‐leader from *S. cerevisae*; α^PcL^, evolved α‐factor prepro‐leader from a previous evolution campaign performed on PcL (Camarero *et al*., 2012); α^PM^ ^1L^, evolved α‐factor prepro‐leader from a previous evolution campaign performed on PM1L (Mate *et al*., 2010). Measurements were made in triplicate on supernatants from three independent fermentations, and they are expressed as the mean including standard deviation. A single *S. cerevisiae* colony was picked from the SC dropout plate for each laccase construct, inoculated in minimal SC medium (20 ml) and incubated for 48 h at 30°C and 220 rpm (Minitron‐INFORS, Biogen Spain). An aliquot of cells was used to inoculate minimal SC medium (20 ml) in a 100 ml flask (optical density at 600 nm [OD_600_] 0.25), the cells were allowed to complete two growth phases (6 to 8 h; OD_600_ = 1) and 2 ml of the culture was them added to the laccase expression medium (18 ml) in a 100 ml flask. After incubation for 72 h at 30°C and 220 rpm, the cells were harvested by centrifugation at 4500 rpm and 4°C (Eppendorf 5810R centrifuge, Germany) and supernatants assayed for ABTS activity as described previously. Minimal SC medium contained 100 ml of 6.7% (w/v) sterile yeast nitrogen base, 100 ml of a 19.2 g l^−1^ sterile yeast synthetic dropout medium supplement without uracil, 100 ml of sterile 20% (w/v) raffinose, 700 ml of *sdd*H_2_O and 1 ml of chloramphenicol (25 g l^−1^). YP medium contained 10 g of yeast extract, 20 g of peptone and *sdd*H_2_O to 650 ml. Laccase expression medium contained 144 ml of 1.55xYP, 13.4 ml of 1 M KH_2_ PO_4_ (pH 6.0) buffer, 22.2 ml of 2% (w/v) galactose, 0.4 ml CuSO_4_ (1M), 0.200 ml of chloramphenicol (25 g l^−1^) and *sdd*H_2_O to 200 ml.

Given that the growth conditions in the HTS/SEM experiments were restricted (i.e. OD_600_ < 1), to fully analyse the effects of evolved prepro‐leaders on secretion while circumventing possible metabolic/culture burdens, the ensemble of laccase fusions were tested in flask fermentations with rich medium (with OD_600_ ~35–40), Fig. 3. Under these conditions, evolved prepro‐leaders outperformed the secretion achieved by α^native^, no matter the laccase attached. This was especially conspicuous for high‐redox potential laccases, the secretion of which increased up to ~50‐fold when they were associated to α^PM1L^ or α^PcL^, Fig. 3A. By contrast, laccase cultures with SEM in flask followed a similar secretion pattern as that obtained in HTS/SEM experiments but they were precluded for larger scale production due to the limited growth of yeast in SEM (with OD_600_ < 15). Thus, the composition of the medium, the format and the culture conditions become key drivers when assessing laccase activity, such that the secretion observed in HTS format within a directed evolution experiment cannot always be extrapolated to large fermentations.

Both the α^PcL^ and α^PM1L^ evolved prepro‐leaders are derived from several rounds of directed evolution to enhance the secretion of PcL and PM1L, and they share common features. First, a similar mutation was introduced independently in the canonical pre‐leader of each signal peptide (A9D and V10D for α^PcL^ and α^PM1L^, respectively, see Fig. 1). These mutations are located in the hydrophobic domain of the pre‐region that is involved in ER targeting. In our previous studies, we showed that, individually, these mutations improve the secretion of their fused laccase sequences by reducing markedly hydrophobicity during the extrusion of the polypeptide laccase chain into the bilayer of the ER, while their combination did not benefit secretion (Mate *et al*., 2010; Camarero *et al*., 2012). In addition to the V10D mutation, α^PM1L^ contains a mutation in one of the three sites for N‐linked glycosylation of the pro‐leader (N23K, within the Asn‐X‐Ser/Thr recognition motif). Similarly, α^PcL^ carries the S58G mutation located in the second N‐glycosylation site and although in this case the glycosylation site was not lost, it seems plausible that its affinity for sugar anchoring might have changed. The effect of such substitutions on secretion remains uncertain; however, a similar change at the third glycosylation site (N57D) was also reported in the best evolved α‐factor prepro‐leader appS4 that improved antibody secretion, reflecting the possible role that these three glycosylation sites could have on exocytosis (Rakestraw *et al*., 2009). The F48S of α^PcL^ is another mutation located at the pro‐leader. A similar substitution (F48/S/V) was again observed in four leaders evolved for antibodies secretion, which highlights how this mutation enhances the secretion of a variety of proteins, even those from quite distant families. Finally, the mutations E86G and A87T respectively found in α^PcL^ and in α^PM1L^ modify the STE13 processing site which, in turn, could affect the performance of KEX2 in the Golgi compartment during the final maturation stages.

## Conclusions

We describe here the use of evolved α‐factor prepro‐leaders for the functional expression in *S. cerevisiae* of fungal laccases with different redox potentials to perform directed evolution experiments. When we tested such prepro‐leaders within a HTS context, assaying different redox mediators, their secretion was mainly related to the laccase sequences from which they were evolved. By contrast, in flask fermentations with rich medium the evolved signal sequences improved secretion regardless of the laccase attached, taking one step closer to their ‘universality’ at least within the laccase enzyme group. These evolved leaders share certain similarities with other α‐factor prepro‐leaders evolved to express proteins from different sources, which opens a new avenue to engineer universal signal peptides for expression in yeast.

## Conflict of Interest

None declared.

## References

[mbt212838-bib-0001] Alcalde, M. (2007) Laccases: biological functions, molecular structure and industrial applications In Industrial Enzymes. Structure, Function and Applications. Polaina J., and MacCabe A.P. (eds). Dordrecht: Springer, pp. 461–476.

[mbt212838-bib-0002] Alcalde, M. (2010) Mutagenesis protocols in *Saccharomyces cerevisiae* by in vivo overlap extension In In Vitro Mutagenesis Protocols, 3rd edn *Methods in Molecular Biology* 634. Bramman J. (ed). Totowa, NJ: Springer‐Humana Press, pp. 3–15. 10.1007/978-1-60761-652-8_1 20676972

[mbt212838-bib-0003] Alcalde, M. (2015) Engineering the ligninolytic enzyme consortium. Trends Biotechnol 33: 155–162. 25600621 10.1016/j.tibtech.2014.12.007

[mbt212838-bib-0004] Bulter, T. , Alcalde, M. , Sieber, V. , Meinhold, P. , Schlachtbauer, C. , and Arnold, F.H. (2003) Functional expression of a fungal laccase in *Saccharomyces cerevisiae* by directed evolution. Appl Environ Microbiol 69: 987–995. 12571021 10.1128/AEM.69.2.987-995.2003 PMC143632

[mbt212838-bib-0005] Camarero, S. , Pardo, I. , Cañas, A.I. , Molina, P. , Record, E. , Martínez, A.T. , *et al* (2012) Engineering platforms for directed evolution of laccase from *Pycnoporus cinnabarinus* . Appl Environ Microbiol 78: 1370–1384. 22210206 10.1128/AEM.07530-11 PMC3294479

[mbt212838-bib-0006] Cañas, A.I. , and Camarero, S. (2010) Laccases and their natural mediators: biotechnological tools for sustainable eco‐friendly processes. Biotechnol Adv 28: 694–705. 20471466 10.1016/j.biotechadv.2010.05.002

[mbt212838-bib-0007] Dana, C.M. , Saija, P. , Kal, S.M. , Bryan, M.B. , Blanch, H.W. , and Clark, D. (2012) Biased clique shuffling reveals stabilizing mutations in cellulase Cel7A. Biotechnol Bioeng 109: 2710–2719. 22887329 10.1002/bit.24708

[mbt212838-bib-0008] Gianfreda, L. , Xu, F. , and Bollag, J. (1999) Laccases: a useful group of oxidoreductive enzymes. Bioremediat J 3: 1–25.

[mbt212838-bib-0009] Gonzalez‐Perez, D. , Garcia‐Ruiz, E. , and Alcalde, M. (2012) *Saccharomyces cerevisiae* in directed evolution: an efficient tool to improve enzymes. Bioengineered 3: 1–6. 10.4161/bbug.19544 PMC3370936 22572788

[mbt212838-bib-0010] Klonowska, A. , Gaudin, C. , Asso, M. , Fournel, A. , Reglier, M. , and Tron, T. (2005) LAC3, a new low redox potential laccase from Trametes sp. Strain C30 obtained as a recombinant protein in yeast. Enzyme Microb Technol 36: 34–41.

[mbt212838-bib-0011] Kunamneni, A. , Plou, F.J. , Ballesteros, A. , and Alcalde, M. (2008a) Laccases and their applications: a patent review. Recent Pat Biotechnol 2: 10–24. 19075849 10.2174/187220808783330965

[mbt212838-bib-0012] Kunamneni, A. , Camarero, S. , Garcia, C. , Plou, F.J. , Ballesteros, A. , and Alcalde, M. (2008b) Engineering and applications of fungal laccases for organic synthesis. Microb Cell Fact 7: 32. 19019256 10.1186/1475-2859-7-32 PMC2613868

[mbt212838-bib-0013] Mate, D.M. , and Alcalde, M. (2015) Laccase engineering: from rational design to directed evolution. Biotechnol Adv 33: 25–40. 25545886 10.1016/j.biotechadv.2014.12.007

[mbt212838-bib-0014] Mate, D.M. , and Alcalde, M. (2017) Laccase: a multi‐purpose biocatalyst at the forefront of biotechnology. Microb Biotechnol https://doi.org/10.1111/1751-7915.12422 10.1111/1751-7915.12422 PMC5658592 27696775

[mbt212838-bib-0015] Mate, D.M. , and Alcalde, M. (2016) Directed evolution of fungal laccases: an update In Advances in Genome Science, Vol 4. Rahman A.U. (ed.). Sharjah U.A.E.: Bentham Science Publisher, pp. 91–112.

[mbt212838-bib-0016] Mate, D. , García‐Burgos, C. , García, E. , Ballesteros, A. , Camarero, S. , and Alcalde, M. (2010) Laboratory evolution of high redox potential laccases. Chem Biol 17: 1030–1041. 20851352 10.1016/j.chembiol.2010.07.010

[mbt212838-bib-0017] Molina‐Espeja, P. , Garcia‐Ruiz, E. , Gonzalez‐Perez, D. , Ullrich, R. , Hofrichter, M. , and Alcalde, M. (2014) Directed evolution of unspecific peroxygenase from *Agrocybe aegerita* . Appl Environ Microbiol 80: 3496–3507. 24682297 10.1128/AEM.00490-14 PMC4018863

[mbt212838-bib-0018] Morozova, O.V. , Shumakovich, G.P. , Shleev, S.V. , and Yaropolov, Y.I. (2007) Laccase‐mediator systems and their applications: a review. Appl Biochem Microbiol 43: 523–535. 18038679

[mbt212838-bib-0019] Rakestraw, J.A. , Sazinsky, S.L. , Piatesi, A. , Antipov, E. , and Wittrup, K.D. (2009) Directed evolution of a secretory leader for the improved expression of heterologous proteins and full‐length antibodies in *Saccharomyces cerevisiae* . Biotechnol Bioeng 103: 1192–1201. 19459139 10.1002/bit.22338 PMC2847895

[mbt212838-bib-0020] Riva, S. (2006) Laccases: blue enzymes for green chemistry. Trends Biotechnol 24: 219–226. 16574262 10.1016/j.tibtech.2006.03.006

[mbt212838-bib-0021] Rochefort, D. , Leech, D. , and Bourbonnais, R. (2004) Electron transfer mediator systems for bleaching of paper pulp. Green Chem 6: 14–24.

[mbt212838-bib-0022] Rodgers, C.J. , Blanford, C.F. , Giddens, S.R. , Skamnioti, P. , Armstrong, F.A. , and Gurr, S.J. (2010) Designer laccases: a vogue for high‐potential fungal enzymes? Trends Biotechnol 28: 63–72. 19963293 10.1016/j.tibtech.2009.11.001

[mbt212838-bib-0023] Romanos, M.A. , Scorer, C.A. , and Clare, J.J. (1992) Foreign gene expression in yeast: a review. Yeast 8: 423–488. 1502852 10.1002/yea.320080602

[mbt212838-bib-0024] Shuster, J.R. (1991) Gene expression in yeast: protein secretion. Curr Opin Biotechnol 2: 685–690. 1367718 10.1016/0958-1669(91)90035-4

[mbt212838-bib-0025] Solomon, E.I. , Sundaram, U.M. , and Machonkin, T.E. (1996) Multicopper oxidases and oxygenases. Chem Rev 96: 2563–2605. 11848837 10.1021/cr950046o

